# CXCR3 Signaling in BRAF^WT^ Melanoma Increases IL-8 Expression and Tumorigenicity

**DOI:** 10.1371/journal.pone.0121140

**Published:** 2015-03-23

**Authors:** Molly H. Jenkins, Constance E. Brinckerhoff, David W. Mullins

**Affiliations:** 1 Department of Medicine, Geisel School of Medicine at Dartmouth, Lebanon, New Hampshire, United States of America; 2 Department of Microbiology and Immunology, Geisel School of Medicine at Dartmouth, Lebanon, New Hampshire, United States of America; 3 Department of Biochemistry, Geisel School of Medicine at Dartmouth, Lebanon, New Hampshire, United States of America; 4 Norris Cotton Cancer Center, Geisel School of Medicine at Dartmouth, Lebanon, New Hampshire, United States of America; Rutgers University, UNITED STATES

## Abstract

Patients with early stage, radial growth phase (RGP) melanoma have a 97% survival rate; however, when the melanoma progresses to the invasive vertical growth phase (VGP), survival rates decrease to 15%. The targets of many clinical trials are the known genetic and molecular mechanisms involved in melanoma progression, with the most common oncogenic mutation being the BRAF^V600E^. However, less than half of melanomas harbor this mutation, and consequently, do not respond to the current BRAF targeted treatments. It is therefore critical to elucidate alternative mechanisms regulating melanoma progression. Increased expression of the chemokine receptor, CXCR3, on melanoma cells is correlated with increased metastasis and poor patient outcomes, suggesting a role for CXCR3 in the RGP to VGP transition. We found that endogenous CXCR3 can be induced in two RGP cell lines, BOWES (BRAF^WT^) and WM35 (BRAF^V600E^), with *in vitro* environmental stress and nutrient deprivation. Signaling via induced endogenous CXCR3 is linked with IL-8 expression in BOWES cells. Ectopic overexpression of CXCR3 in BOWES cells leads to increased ligand-mediated phERK, cellular migration, and IL-8 expression *in vitro*, and to increased tumorigenesis and lymph node metastasis *in vivo*. Our results demonstrate that, in BRAF^WT^ melanomas, CXCR3 signaling mediates significant increases in IL-8 expression, suggesting that CXCR3 expression and signaling may represent a transformative event that drives the progression of BRAF^WT^ melanomas. **Implications:** Expression of CXCR3 on BRAF^WT^ melanoma cells may be a mediator of melanoma progression.

## Introduction

Melanoma progression has been described as a multi-step process, with melanocytes changing to nevi, and then primary melanoma with radial growth phase (RGP) transitioning to vertical growth phase (VGP) [1–3]. Early stage RGP melanomas are confined to the epidermis and readily curable by surgical excision; however, melanomas acquire increased invasive and metastatic potential as they progress to VGP, and patients with VGP melanoma are often resistant to treatment. The transition from RGP to VGP melanoma is a critical step in melanoma progression, with 5-year survival rates decreasing from 97% (RGP patients) to 15% (VGP patients) [4–8], making it critical to elucidate the molecular changes associated with this transition.

The targets of many current clinical trials are known genetic and molecular mechanisms involved in melanoma progression, with the most common oncogenic mutation being the BRAF^V600E^ (found in nearly 50% of melanoma) [9–12]. However, melanoma can progress from RGP to VGP in the absence of this mutation (i.e. wild-type BRAF) [13], suggesting that this mutation is not the only factor mediating melanoma invasion and metastasis. In addition, since less than half of melanomas harbor the BRAF^V600E^ mutation, and consequently, do not respond to the current targeted treatment, it is critical to delineate alternative mechanisms that regulate the progression of melanoma.

Known mediators of melanoma progression include matrix metalloproteinases (MMPs), cytokines, and oncogenes [14–18]. MMPs are important in matrix degradation and remodeling, a process that needs to occur in the skin before RGP melanoma cells can break through the basement membrane and acquire an invasive, VGP phenotype. Increased expression of MMP-1, MT1-MMP, and MMP-2 have been correlated with increased invasion and metastasis of human melanoma [14–16]. Melanoma cells often express various cytokines and their receptors at different stages of disease progression. IL-1β, IL-6, and IL-8, found in the melanoma tumor microenvironment, are important drivers of cell proliferation and melanoma progression [17,18]. One study, which attempted to identify a molecular signature in human RGP and VGP melanoma cell lines, found an invasion-specific signature that was characterized by the upregulation of genes including *IL-6*, *IL-8*, and *MMP-1* [19]. Another study demonstrated that expression of IL-8 in RGP melanoma cells significantly increased their tumorigenicity and metastatic potential [20].

Although the chemokine receptor, CXCR3, is normally expressed on activated lymphocytes [21] and involved in directing their migration to damaged tissue [22], it is also expressed on many human and murine cancer cells [23–25]. High CXCR3 expression in human VGP melanoma [23,26] correlates with increased metastasis and poor patient outcomes [25], suggesting that CXCR3 signaling may be associated with the RGP to VGP transition. As tumors expand, melanoma cells are exposed to increasing cellular stress, such as hypoxia and nutrient deprivation [27]. Increased expression of surface CXCR3 protein has been correlated with hypoxia and nutrient deprivation *in vitro* in human breast [28] and colon [24] cancer cell lines, suggesting that cells expressing CXCR3 can survive and grow in the less favorable microenvironments of advanced cancer (i.e., VGP melanoma).

In this study, we demonstrate that signaling via CXCR3 on a human RGP BRAF^WT^ cell line (BOWES) is linked with IL-8 expression. Ectopic overexpression of CXCR3 in these BOWES cells leads to increased ligand-mediated phosphorylation of ERK and cellular migration *in vitro*, and to increased tumorigenesis and metastasis *in vivo*. While endogenous CXCR3 can also be induced with stress in WM35 cells, a human RGP BRAF^V600E^ cell line, we found that IL-8 expression in these cells was not linked with CXCR3 signaling. These results suggest that CXCR3 signaling may mediate IL-8 expression in RGP cells, independent of constitutive BRAF signaling, and that CXCR3 may be a driver in melanoma progression in BRAF^WT^ melanoma patients.

## Materials and Methods

### Cell lines

BOWES cells were obtained from the American Type Cell Culture (ATCC, Manassas, VA [15]) and cultured in EMEM (ATCC) containing 10% FBS, HEPES (Fisher Bioreagents, Pittsburgh, PA), and 1x penicillin/streptomycin (Corning Inc., Corning, NY). WM35 cells were obtained from Meenhard Herlyn’s laboratory at The Wistar Institute and were grown in DMEM/F-12 advanced media (Life Technologies, Carlsbad, CA) with 5% FBS, 1x penicillin/streptomycin, and 1x glutamine. Human VGP cell lines (VMM12, VMM5) and clonal lines (VMM5-derived C4, and C9 [29]) were grown in RPMI with 10% FBS and 1x penicillin/streptomycin.

### Exogenous expression of CXCR3

Cells were transfected with a human CXCR3 ORF construct (Origene, Rockville, MD, USA) (under control of the CMV promoter, with Neomycin resistance) or a pCMV6 empty vector control (Origene), using Lipofectamine 3000 (Life Technologies), according to the manufacturer’s instructions. Transfected cells were grown with 500μg/ml G418 (Corning) and plated at a clonal density to yield stable clones.

### Flow cytometry

To measure levels of CXCR3, cells were plated (3 x 10^5^ cells per well) in 6-well plates and allowed to incubate overnight in normal serum-containing culture conditions at 5% CO_2_. The next day, cells were washed 3 times in HBSS to remove serum, and then either incubated with serum-containing media at 5% CO_2_ or serum-free media at 8% CO_2_. After the designated time course (24, 48, or 72 hrs), the cells were washed with HBSS and harvested with Cell Stripper (Corning). Single cell suspensions were stained with APC mouse anti-human CD183 (CXCR3) (BD Biosciences, San Jose, CA, USA) or APC mouse IgG isotype control antibody (Biolegend, San Diego, CA, USA) at a 1:10 dilution using a standard flow cytometry staining protocol. Flow cytometry was performed on a BD FACSCalibur, and analysis was performed using FlowJo analysis software (Ashland, OR).

To sort induced CXCR3^Low^ and CXCR3^High^ populations, cells were plated (3–4 x 10^6^ cells per plate) on 150 mm plates and allowed to incubate overnight in normal serum-containing culture conditions at 5% CO_2_. The next day, cells were washed 3 times in HBSS to remove serum, and then incubated in serum-free media at 8% CO_2_. After 48–72 hrs, the cells were harvested and stained, as above, sorted into RNAlater (Life Technologies) using a BD FACSAria, then spun down and RNA was prepped for RT-PCR.

### RT-PCR analysis

Total RNA was extracted from cells or tumor tissue using an RNeasy kit (Qiagen, Valencia, CA) and reverse transcription was performed using an iScript cDNA Synthesis kit (Bio-Rad, Hercules, CA). Cellular levels of IL-8, HIF-1alpha, IL-1beta, IL-6, MCP-1, Par-1, and MMP-1, -2, -3, -9, -13, and -14 mRNA were measured by qRT-PCR using iQSyber Green Supermix (BioRad) on a CFX96 Real Time System C1000 Thermal Cycler. All samples were normalized to B_2_M and relative fold change was calculated as 2^-ΔΔCt^. Primer sequences are listed in S1 Table. Cellular levels of CXCL10, CXCR3, and IL-8 were measured by qRT-PCR using Taqman Fast Advanced Master Mix (Life Technologies) and Taqman primers (CXCL10—Hs01124251_g1; CXCR3—Hs01847760_s1; IL-8—Hs00174103_m1; GAPDH—Hs02758991_g1 [Life Technologies]) on a StepOnePlus real-time PCR system (Applied Biosystems, Foster City, CA). All samples were normalized to GAPDH and relative fold change was calculated as 2^-ΔΔCt^.

### Immunoblotting

BOWES PCMV6 and BOWES CXCR3 cells were plated (2.5 x 10^5^ cells per well) in 6-well plates and allowed to incubate for 24 hrs in normal serum-containing culture conditions at 5% CO_2_. Cells were then washed 3 times in HBSS to remove serum and incubated in serum-free media at 5% CO_2_ for 2 hrs. CXCR3 ligands, CXCL9 and CXCL10, (100ng/ml each) (Peprotech, Rocky Hill, NJ) were then added to each well and allowed to incubate for 2, 5, 10, 20, or 30 minutes. Total cell lysates (2–5μg) were prepared with RIPA buffer (Sigma Aldrich, St. Louis, MO) and separated on 10% SDS-PAGE gels (Bio-Rad). Standard immunoblotting techniques were used [29,30], and protein was visualized with anti-pERK (9101) or total ERK (9102) (Cell Signaling Technology, Beverly, MA).

### ELISA

To assess the concentration of CXCL10 in NSG mice, human melanoma cell lines were xenotransplanted in NSG mice. When the tumors reached 10 mm in diameter, the mice were sacrificed and tumor tissue and spleens were harvested from individual mice. Tissue was then pressed through a 70 μm filter and lysed in TPER buffer. The concentration of mouse CXCL10 in these tumor and spleen suspensions was then evaluated by a mouse CXCL10 ELISA kit (R&D Systems, Minneapolis, MN), per manufacturer’s instructions.

### Migration Assay

Migration of BOWES PCMV6 and BOWES CXCR3 cells was measured using HTS Fluoroblok inserts with 8 -μm pores in a 24-well companion plate (Becton Dickinson). For each assay, serum-free media was loaded into the bottom of the plate with either no ligand, or 200 ng/mL CXCL9 and CXCL10 (Peprotech). Cells (2 x 10^5^) were plated on the insert in serum-free media with either 0 or 1μM of the CXCR3 antagonist AMG487 (MedChemExpress LLC, Princeton, NJ). Plates were incubated at 5% CO_2_ for 6 hrs and then stained with Calcein AM, cell-permeate dye (Life Technologies), for 30 minutes. An Olympus IX50 inverted microscope was used to count the number of fluorescent cells per 40x field. 20 fields were chosen at random from each well and averaged.

### AMG487, U0126, and PLX4032 treatments

Cells (2.5 x 10^5^) were plated in 6-well plates, incubated overnight in serum-containing media, then washed in HBSS. Serum-free media with either DMSO (control), 0.2μM, or 1μM AMG487 was added, with or without 100 ng/mL CXCL9 and CXCL10. RNA was harvested at 48 hrs post AMG487 addition and subsequent RT-PCR analysis of IL-8 was performed. Effects of AMG487 on IL-8 expression in the presence of a MEK inhibitor were assessed using 3μM U0126 (Cell Signaling Technology). Effects of AMG487 on IL-8 expression in the presence of BRAF*V600E* inhibition were evaluated by adding 3μM PLX4032 (ChemiTek, Indianapolis, IN).

### Intradermal injections

Host NOD/SCID/γ chain^null^ (NSG) mice used in this study were obtained from the Transgenic and Genetic construct Mouse Resource Service at Dartmouth College and the Jackson Laboratory (Bar Harbor, Maine). BOWES PCMV6 and BOWES CXCR3 cells were injected intradermally (5 x 10^5^ cells, 50μl HBSS) into male NSG mice into the right flank, 16 mice per group. Mice were examined weekly until tumors were apparent, then the tumor was measured once a week. Each tumor was measured twice with Vernier calipers (Fisher Scientific) and tumor volume was calculated using the formula (4/3)πr^3^. When the two measurements differed, the smaller radius measurement was squared and multiplied by the largest radius measurement. This number was then substituted for the r^3^ portion of the formula [31]. After 6 weeks, when the tumors reached 8–10 mm in diameter, mice were sacrificed by inhalation of isofluorane and cervical dislocation, and tumors and draining lymph nodes were resected from each mouse. All animal procedures were reviewed and approved by the Institutional Animal Care and Use Committee at Dartmouth College.

### *Alu* PCR analysis

DNA was extracted from draining lymph nodes harvested from mice injected with either BOWES PCMV6 or BOWES CXCR3 cells, using DNeasy Blood and Tissue kit (Qiagen, Valencia, CA), following manufacturer’s directions. RT-PCR to amplify the human repetitive sequence *AluI* was performed on 100 ng of tissue DNA using iQSyber Green Supermix (BioRad) on a CFX96 Real Time System C1000 Thermal Cycler RT-PCR, as previously described [32]. The pg of Alu per 100 ng lymph node DNA was calculated and compared to background levels (Alu sequence found in 100ng mouse genomic DNA). Tissue samples that had >0.1pg of Alu more than background levels were considered to have metastases. Data are presented as the number of metastases found in lymph node tissue over the total number of tissues analyzed. A Fisher exact test was used to analyze the data. Primer sequences are listed in S1 Table.

### Statistical analysis

Unless indicated, the Student t test and was used to assess statistical significance using GraphPad Prism software. Linear regression analysis, means, standard deviations and standard errors were calculated using GraphPad Prism software.

## Results

### Endogenous expression of CXCR3 in human melanoma cell lines

**Fig 1:**
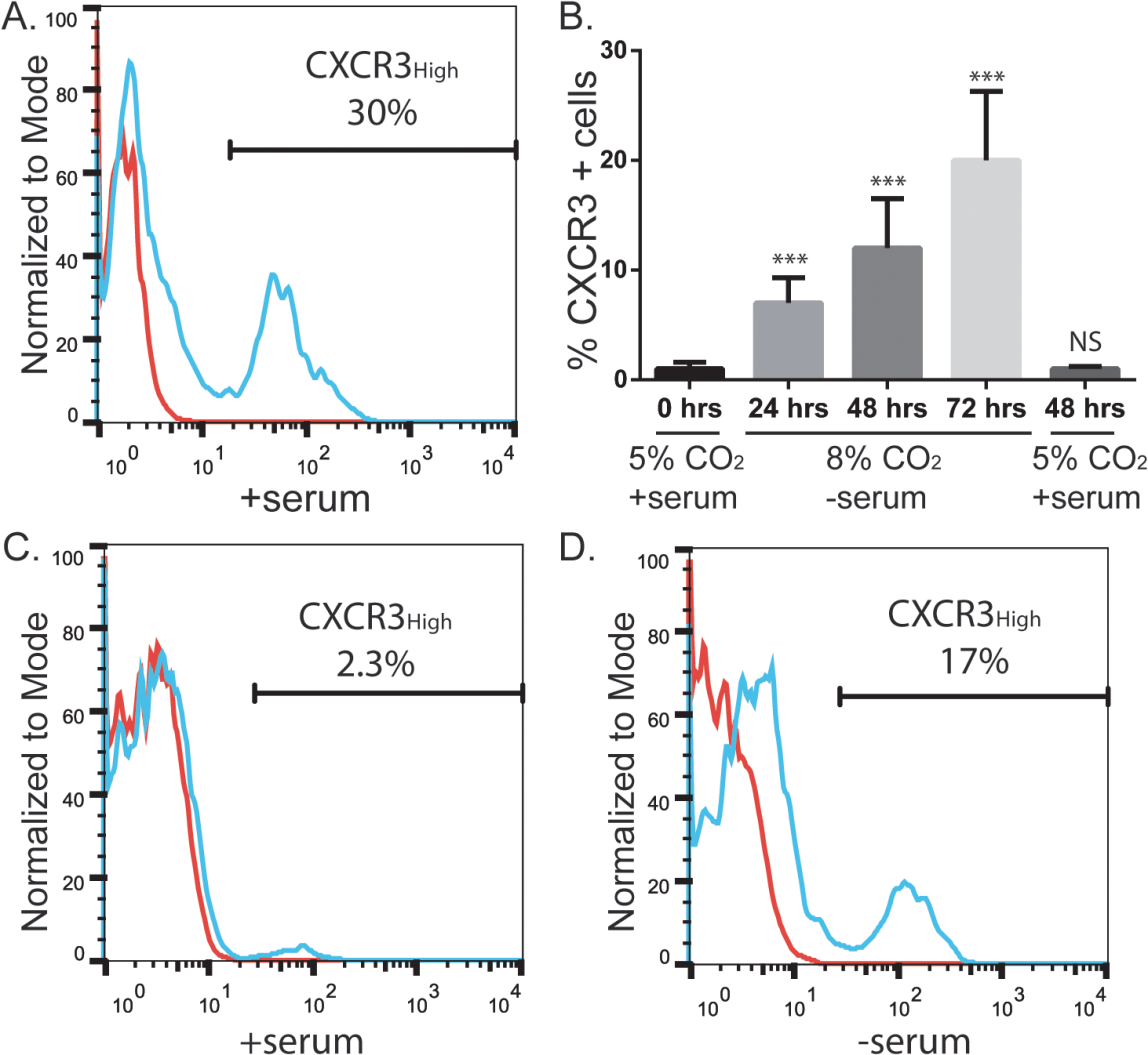
Stress induces CXCR3 expression on BOWES RGP melanoma cells. (A) Flow cytometry analysis of CXCR3 protein expression on a VGP melanoma cell line, C9. Cells were incubated with serum at 5% CO_2_. (B) Graph of CXCR3 protein expression on BOWES cells from 8 separate flow cytometry experiments. Cells were cultured either in normal culture conditions (far left bar), in serum-free media at 8% CO_2_ for 24, 48, or 72 hrs, or in serum-free media at 8% CO_2_ for 48 hrs followed by 48 hrs in normal culture conditions (far right bar). T-tests were performed on the averages for each time point, comparing treatment cells with the 0hr time point (NS = P>0.05, *** = P≤ 0.001). (C-D) Flow cytometry analysis of CXCR3 protein expression on an RGP cell line, BOWES. Cells were incubated (C) with serum at 5% CO_2_ or (D) without serum at 8% CO_2_ for 72 hrs. All flow cytometry plots and graphs are representative data from at least 3 separate experiments. Red lines = unstained cells, blue lines = cells stained with CXCR3.

We used flow cytometry to determine CXCR3 surface protein levels in human melanoma cell lines grown under normal culture conditions (media with 10% fetal bovine serum, incubated at 37°C with 5% CO_2_). We found notable but variable (5–40%) expression of CXCR3 on the four human VGP melanoma cell lines tested; VMM12, VMM5, and two VMM5 clones, C4 and C9 (Fig. 1A and S1 Fig.). These levels are similar to those seen in other VGP lines [25]. In contrast, there was minimal to no expression of surface CXCR3 in BOWES cells, a human BRAF^WT^ RGP cell line, cultured under normal conditions (Fig. 1B and 1C). Similar to human breast and colon cancer cell lines [24,28], we found that growing BOWES cells under conditions of cellular stress (media without serum, incubated at 37°C with 8% CO_2_, hereafter referred to as stressed cultures) increased the proportion of cells expressing CXCR3 (Fig. 1C and 1D). The proportion of CXCR3-expressing cells increased over time, up to 72hrs (Fig. 1B). However, this increased expression was transient, and CXCR3 levels returned to baseline when cells were re-cultured in non-stressed conditions (Fig. 1B).

### Signaling via CXCR3 induces IL-8 expression

To investigate the downstream consequences of stress-induced CXCR3 expression, gene expression was compared between RGP BOWES cells cultured under normal and stress conditions. RT-PCR was performed on the normal and stressed cells using a panel of genes associated with melanoma progression and metastases, including cytokines and matrix metalloproteinases (MMPs) [14–18] (Table 1). Mild (1–10 fold) to no increases were seen in the majority of genes tested, however, there was a significant increase (P<0.0001) in interleukin-8 (IL-8) expression in stressed BOWES cells compared to normal cultured controls (Table 1).

**Table 1:** Gene expression of stressed BOWES cells (cultured in serum-free media at 8% CO_2_) relative to those cultured under normal serum-containing conditions (10% FBS media at 5% CO_2_).

CXCR3	**+**	Par-1	**NC**
CXCL10	**+**	MMP-1	**NC**
HIF-1alpha	**+**	MMP-2	**+**
IL-1beta	**++**	MMP-3	**NC**
IL-6	**++**	MMP-9	**+**
IL-8	**+++**	MMP-13	**NC**
MCP-1	**NC**	MMP-14	-

These experiments were performed without the addition of exogenous ligands.

Changes between normal and stressed culture conditions

NC = no change

- = Decreased expression

+ = 1–10 fold increase

++ = 10–20 fold increase

+++ = up to 20^+^ fold increase

**Fig 2:**
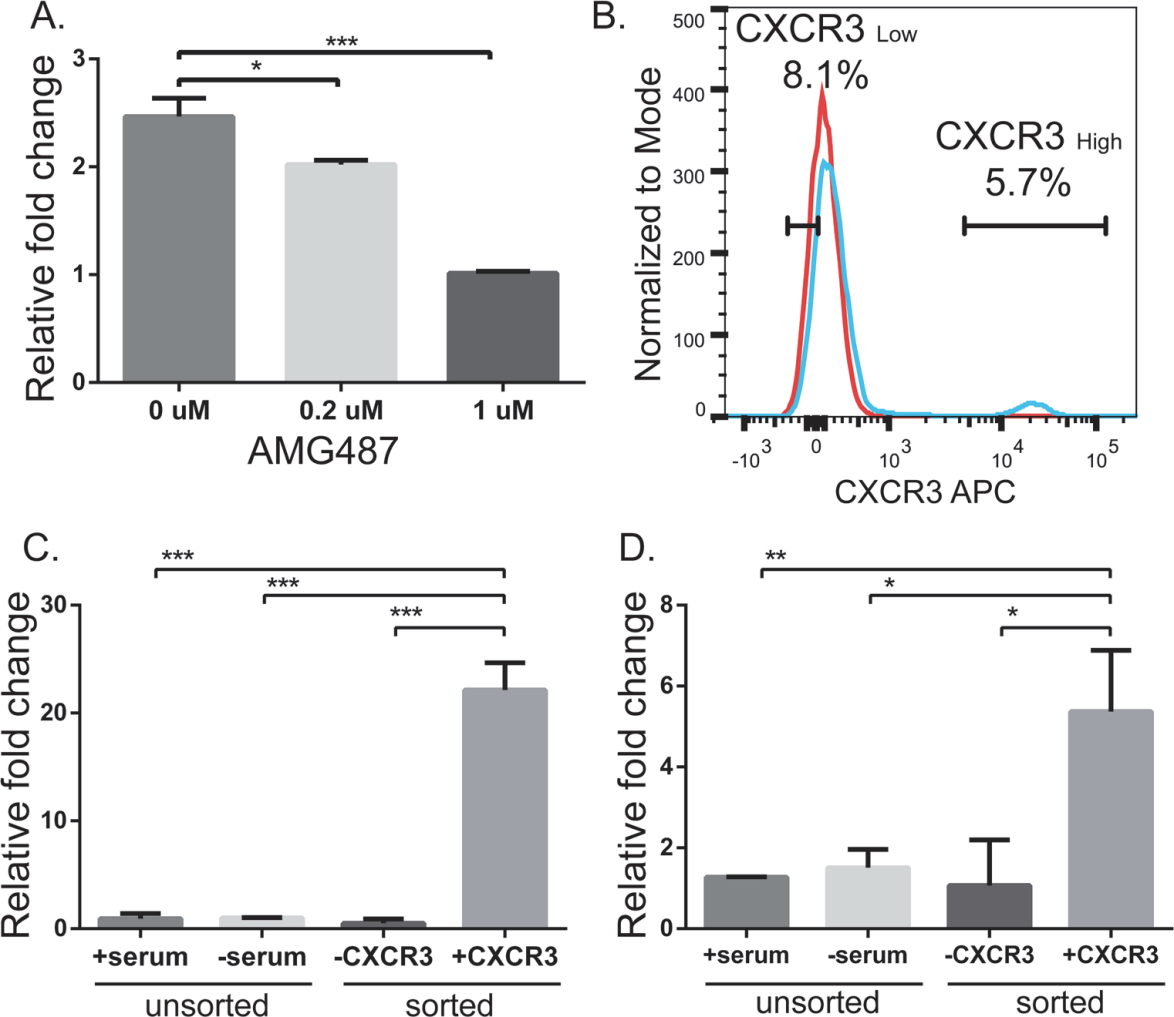
IL-8 expression in stressed BOWES cells is associated with CXCR3 signaling and increased in the CXCR3^High^ subpopulation of cells. (A) BOWES cells were cultured for 48 hrs under stressful conditions (serum-free media at 8% CO_2_) in the presence of ligand (100ng/ml CXCL9 and CXCL10), with the addition of DMSO, 0.2μM, or 1μM AMG487. IL-8 expression was measured with RT-PCR, fold change was calculated relative to cells treated 1μM AMG487. (B) Flow cytometry analysis of representative data from 3 separate sorts. CXCR3^Low^ and CXCR3^High^ cells were sorted based on the gates shown. Red line = unstained cells, blue line = cells stained with CXCR3. (C) CXCR3 and (D) IL-8 RT-PCR results from unsorted cells grown with serum in 5% CO_2_ or without serum in 8% CO_2_ (stressed), and sorted stressed cells (CXCR3^Low^ and CXCR3^High^). Fold change was calculated relative to unsorted stressed cells. T-tests were performed on the relative fold changes, pooled data from 3 separate experiments (* = P≤ 0.05, ** = P≤0.01, ***P≤ 0.001).

IL-8 was originally identified as a leukocyte chemoattractant, but has since been shown to be involved in melanoma progression [33]. One study has found that ectopic expression of IL-8 in RGP melanoma cells increased their tumorigenicity and metastatic potential [20], and we have previously shown a direct correlation between IL-8 mRNA and protein levels in human melanoma cells [29]. Therefore, to test whether increased IL-8 mRNA expression in stressed BOWES cells is driven by CXCR3 signaling, cells were grown in stressed culture conditions for 48 hrs with AMG487, a small molecular weight antagonist of CXCR3 signaling [34,35], and relative expression of IL-8 was compared to control DMSO-treated cells. AMG487 inhibits CXCR3 signaling by obstructing binding of CXCR3 ligands and has been shown to inhibit *in vitro* CXCR3-mediated cell migration by the CXCR3 chemokines, CXCL9, CXCL10, and CXCL11, and to inhibit lung metastasis in a mouse model of metastatic breast cancer [35]. Treatment of stressed BOWES cells with AMG487 significantly decreased IL-8 expression in a dose dependent manner (Fig. 2A and S2A Fig.).

A CXCR3-CXCL10 autocrine loop has previously been found in breast cancer cell lines *in vitro*, where CXCL10 is secreted by the cancer cells [28]. Similarly, we find a 4-fold increase in CXCL10 levels in BOWES cells cultured under stressful conditions compared to those cultured under normal conditions (S2B Fig.). This induction of endogenous CXCL10 by stressed BOWES cells may explain the apparent ligand-independent effect we see in stressed BOWES cells treated with AMG487 (S2A Fig.). However, in either case (with or without exogenous ligand), the CXCR3 inhibitor was able to knock down signaling, demonstrating the ability of CXCR3 to drive IL-8 expression.

To further investigate the link between IL-8 and CXCR3, BOWES cells were stressed and the subpopulations of CXCR3^High^ and CXCR3^Low^ cells were sorted from bulk stressed cells using florescent activated cell sorting (Fig. 2B). Gene expression analysis of these subpopulations revealed a significant increase in CXCR3 mRNA in the CXCR3^High^ population of cells compared to the CXCR3^Low^ and unsorted populations (Fig. 2C, P≤0.001). In contrast, when we compared unsorted stressed cells to unsorted cells cultured under normal conditions, we found significant (P≤0.05), but highly variable IL-8 expression (Table 1 and Fig. 2D). Fig. 2D, represents pooled data from 3 separate experiments. However, in the sorted populations, we saw a significant and consistent increase in IL-8 mRNA in the CXCR3^High^ population of cells compared to the CXCR3^Low^ population (Fig. 2D, P≤0.05). Thus, these data suggest that the CXCR3^High^ subpopulation is a major contributor to IL-8 expression.

### Ectopic overexpression of CXCR3 in BOWES cells

**Fig 3:**
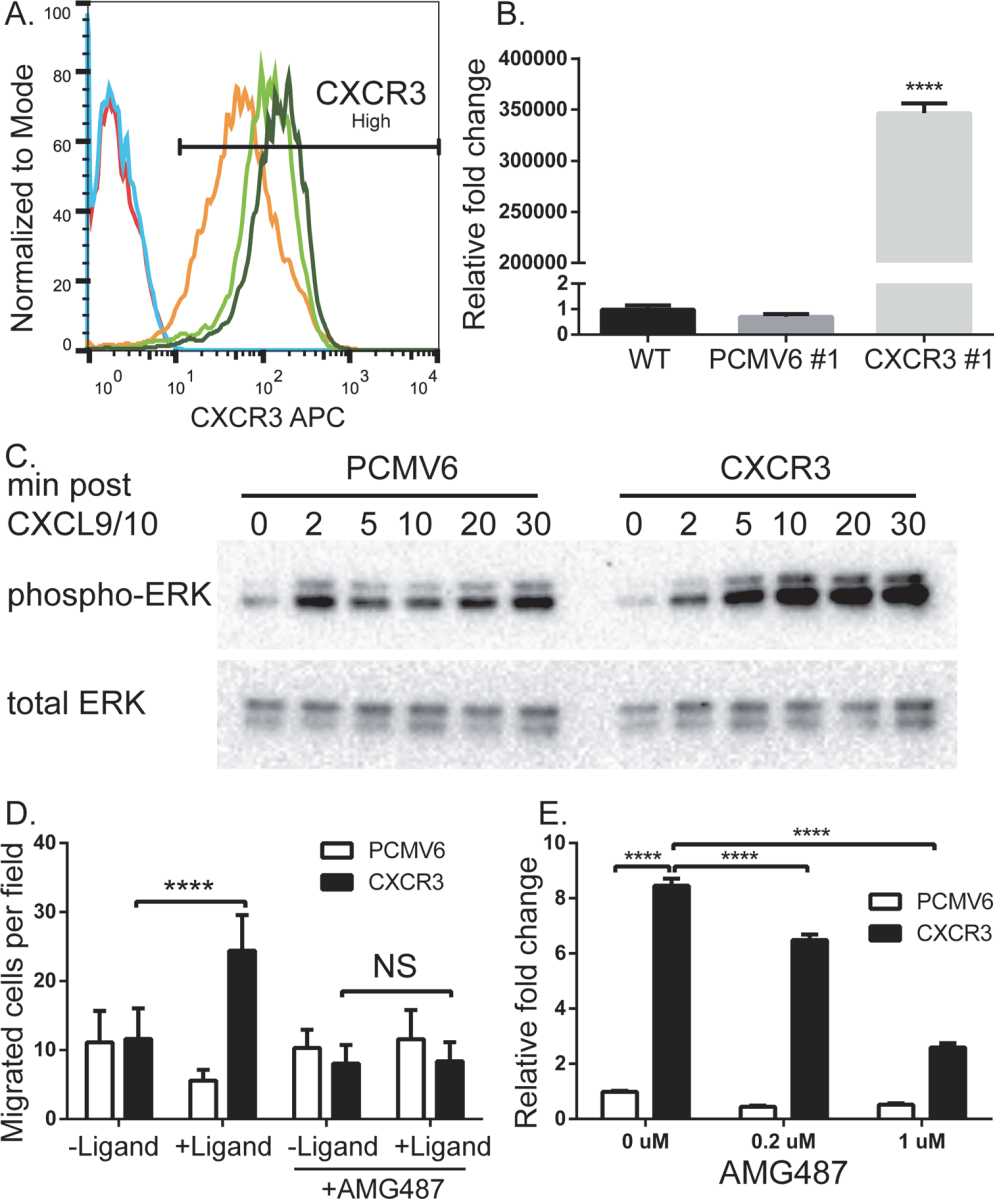
Ectopic overexpression of CXCR3 in BOWES cells increases ligand mediated phosphorylation of ERK, cellular migration, and IL-8 expression. BOWES cells stably transfected with CXCR3 or an empty vector (PCMV6) were grown in serum-containing media. (A) Flow cytometry and (B) RT-PCR were used to measure CXCR3 expression. (A) BOWES WT (red line), BOWES PCMV6 clone #1(blue line), and BOWES CXCR3 clones #1 (orange line), #2 (light green), and #3 (dark green line). (B) RT-PCR was normalized to CXCR3 clone #2. (C) BOWES PCMV6 and CXCR3 cells were grown in serum-free media for 2 hrs, then in the presence of CXCL9 and CXCL10 for 2, 5, 10, 20, or 30 minutes. Total protein was isolated and probed for pERK and total ERK via immunoblot analysis. (D) BOWES PCMV6 and CXCR3 cells were plated on a membrane above serum-free media with CXCL9/10 (+ligand) or without (-ligand), and total migrated cells per field (40X objective) were quantified after 6hrs. Cells were also plated on the membrane in the presence or absence of AMG487. T-tests were performed on the BOWES CXCR3 migrated cells ± ligand in the presence and absence of AMG487 (NS = P>0.05, **** = P≤ 0.0001). (E) BOWES PCMV6 and CXCR3 cells were grown in serum-containing media for 48 hrs in the presence of ligand, with 0, 0.2, or 1 μM AMG487. Expression of IL-8 was measured via RT-PCR, fold change was calculated relative to BOWES PCMV6 0 μM. T-tests were performed on the relative fold changes, representative data from 3 separate experiments (**** = P≤ 0.0001).

To further study the consequences of CXCR3 signaling and the potential role of CXCR3 in the RGP to VGP transition, human CXCR3 was ectopically overexpressed in BOWES cells and stable clones were selected. All three of the BOWES CXCR3 clonal lines expressed greater than 98% CXCR3^High^ cells (Fig. 3A) and had similar mRNA expression (data not shown). In contrast, the isotype control antibody showed no staining in the BOWES CXCR3 cells (S3 Fig.). The BOWES pCMV6 (empty vector) clones and BOWES WT line had very low levels of CXCR3 protein (Fig. 3A) and mRNA, while expression of CXCR3 mRNA was significantly greater in the BOWES CXCR3 clones (Fig. 3B, P≤0.0001).

Signal transduction and migration assays were performed to assess the function of ectopically expressed CXCR3 protein in response to the human CXCR3 ligands, CXCL9 and CXCL10. Phosphorylation of ERK1/2 increased substantially in BOWES CXCR3 cells after ligand addition, relative to BOWES PCMV6 (empty vector transfected) cells, which showed a slight, but transient increase (Fig. 3C). We find that addition of mouse CXCL9 and CXCL10 caused similar increase in the phosphorylation of ERK1/2 in BOWES CXCR3 cells, but not in BOWES PCMV6 cells (data not shown). This agrees with other investigators [36], suggesting that endogenous ligands in a murine host can activate human CXCR3 on cell lines xenograft in mice. We also found abundant murine CXCR3 ligands in the tumor microenvironment of human melanoma cell lines xenotransplanted in NSG mice. CXCL10 levels were 271.4 ± 10 pg/mg in tumor tissue and 74.1 ± 10.9 pg/mg in spleens. These findings demonstrate elevated levels of CXCR3 ligands in the tumor microenvironment of NSG mice, suggesting that NSG mice are an appropriate model to support CXCR3 signaling *in vivo*.

A chemotactic gradient of CXCL9 and CXCL10 resulted in significant increases in the migration of BOWES CXCR3 cells across a membrane compared to no ligand control wells (Fig. 3D, P≤0.0001), and addition of AMG487 to BOWES CXCR3 cells inhibited migration towards CXCL9 and CXCL10 (Fig. 3D, P>0.5). Migration of BOWES PCMV6 cells remained constant regardless of ligand addition or AMG487 treatment. Collectively, these data demonstrate the functionality of exogenously-expressed CXCR3.

IL-8 signaling was then compared between BOWES CXCR3 and PCMV6 cells in the presence of ligand and several doses of AMG487. We found that BOWES CXCR3 cells have significantly more IL-8 expression than BOWES PCMV6 cells in the absence of AMG487 (Fig. 3E, P≤0.0001), but that this expression was significantly decreased upon addition of 0.2 or 1μM AMG487 (P≤0.0001), demonstrating that CXCR3 signaling significantly enhances IL-8 expression.

### CXCR3-induced IL-8 expression is MAPK dependent

**Fig 4:**
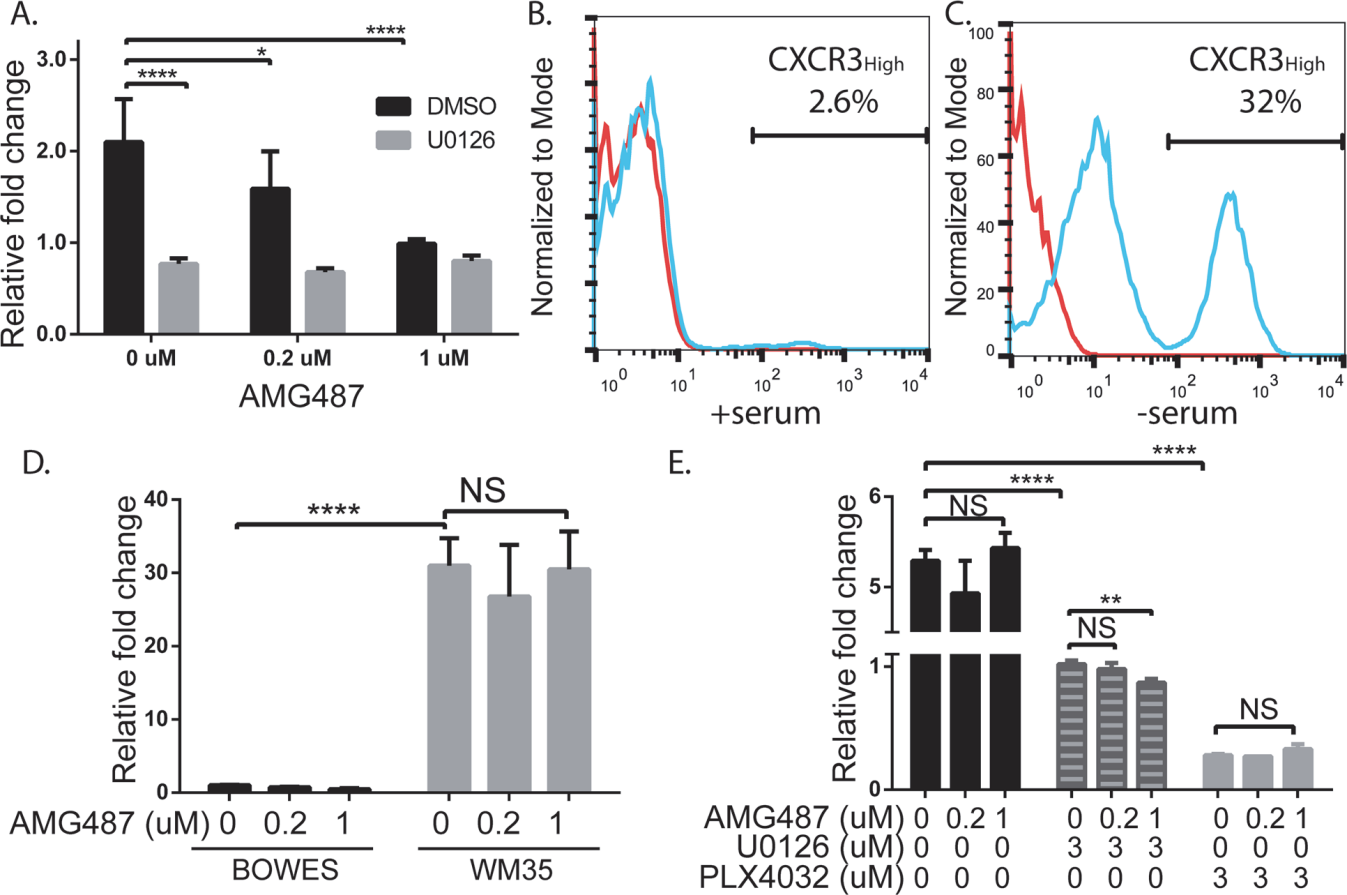
IL-8 expression in BOWES cells is linked with MAPK signaling cascades in BRAF^WT^ BOWES cells. WM35 IL-8 signaling is CXCR3-signaling independent. (A) BOWES WT cells were grown in serum-free media for 48 hrs in the presence of ligand, with 0, 0.2, or 1 μM AMG487 and with DMSO (black bars) or 3 μM U0126 (gray bars). Expression of IL-8 was measured via RT-PCR, fold change was calculated relative to cells treated with 0 μM U0126 and 1 μM AMG487. (B-C) Flow cytometry analysis of CXCR3 protein expression on the BRAF^V600E^ RGP cell line, WM35. Cells were incubated (B) with 5% serum at 5% CO_2_ or (C) without serum at 8% CO_2_ for 72 hrs. Red lines = unstained cells, blue lines = cells stained with CXCR3. (D) BOWES WT (black bars) and WM35 cells (gray bars) were grown in serum-free media for 48 hrs in the presence of ligand, with 0, 0.2, or 1 μM AMG487. Expression of IL-8 was measured via RT-PCR, fold change was calculated relative to BOWES cells treated with 0 μM AMG487. (E) WM35 cells were grown in serum-free media for 48 hrs in the presence of ligand, with 0, 0.2, or 1 μM AMG487 and either DMSO (black bars), 3 μM U0126 (striped bars), or 3 μM PLX4032 (gray bars). Expression of IL-8 was measured via RT-PCR, fold change was calculated relative to WM35 cells treated with 0 μM AMG487 and 3 μM U0126. All T-tests were performed on the relative fold changes, all data is representative from at least 3 separate experiments (NS = P>0.5, * = P≤ 0.05, ** = P≤0.01, **** = P≤ 0.0001).

We next asked whether CXCR3-mediated ERK signaling was necessary for IL-8 expression. BOWES WT cells were stressed for 48hrs in the presence of CXCR3 ligands and either a DMSO control, AMG487 and/or the MEK inhibitor, U0126. Levels of IL-8 significantly decreased in DMSO-treated BOWES cells treated with 0.2μM and 1μM AMG487 (Fig. 4A, P≤0.05 and P≤0.0001, respectively (black bars)). Further, expression of IL-8 was significantly reduced in the presence of 3 μM U0126 (gray bars) compared to DMSO-treated cells (black bars), suggesting that the MEK/ERK pathway facilitates IL-8 expression in these cells. Interestingly, U0126-treated BOWES cells (gray bars) showed no significant change in IL-8 expression with either concentration of AMG487 (P>0.05). These results suggest that stress-induced CXCR3 may increase IL-8 expression through Mitogen Activated Protein Kinase (MAPK) signaling cascades in BRAF^WT^ RGP cells.

Since MAPK signaling cascades are constitutively activated in BRAF^V600E^ cell lines [37–41], but not BRAF^WT^ cell lines, we investigated whether WM35 cells, a BRAF^V600E^ RGP cell line [42], was sensitive to CXCR3 signaling effects. Similar to BOWES cells, WM35 cells show low CXCR3 expression under normal culture conditions (Fig. 4B), but expression is induced under stressed culture conditions (Fig. 4C). Although both RGP cell lines have similar levels of stress-induced CXCR3 regardless of BRAF status, stressed WM35 cells had much higher levels of IL-8 than stressed BOWES cells (Fig. 4D, P≤0.0001), consistent with findings that IL-8 levels are increased in human melanoma cell lines containing the BRAF^V600E^ mutation [43]. However, in contrast to BOWES cells, treatment of WM35 cells with AMG487 did not reduce IL-8 levels (Fig. 4D, gray bars), suggesting that CXCR3 signaling in these cells does not significantly contribute to the high IL-8 levels.

Finally, because inhibition of MAPK signaling reduces IL-8 expression in BRAF^V600E^ cell lines and tumors [43–45], we examined effects of MAPK inhibition on IL-8 expression in WM35 cells. In keeping with these studies, we found reduced IL-8 expression in WM35 cells treated with the MEK inhibitor, U0126 (Fig. 4E, striped bars), and even greater reduction with the BRAF^V600E^ specific inhibitor, PLX4032 (Fig. 4E, gray bars). Interestingly, addition of 1μM AMG487 significantly reduced IL-8 expression in WM35 cells treated with 3μM U0126 (Fig. 4E, striped bars). Collectively, these data demonstrate that CXCR3 signaling mediates IL-8 expression in BRAF^WT^ and BRAF^V600E^ cells. However, in BRAF^V600E^ cells, the contribution of CXCR3 signaling to the total IL-8 production may be inconsequential in the context of constitutive activation of the MAPK pathway.

### *In vivo* consequences of CXCR3 expression

**Fig 5:**
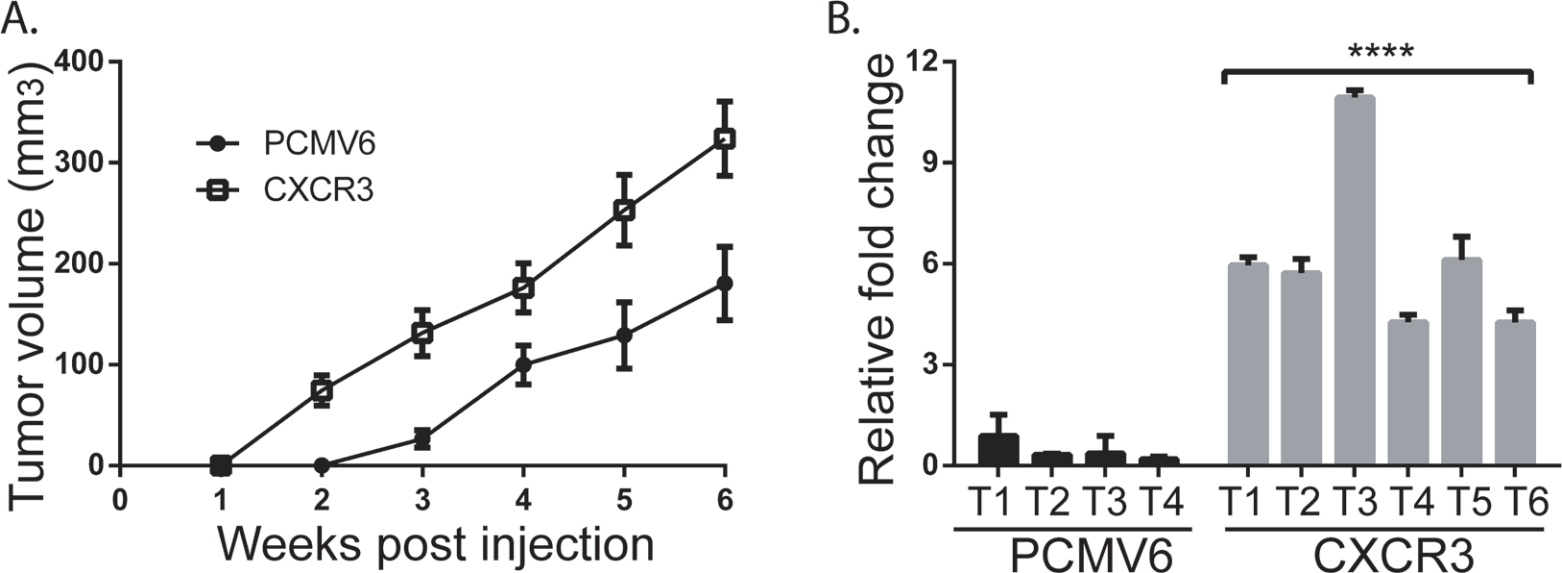
Ectopic overexpression of CXCR3 on BOWES RGP cells increases tumor frequency and metastases and IL-8 expression *in vivo*. BOWES PCMV6 and CXCR3 cells were injected interdermally into NSG mice (5 x 10^5^ cells per injection). Tumors were measured weekly with calipers and tumor volume (mm^3^) was calculated. Mean tumor volume and SEM were calculated from mice that had measurable tumors. (A) The graph shows mean tumor volume (with SEM) over the 6 week period. Linear regression analysis was performed on the two slopes (P = 0.006). (B) Tumor tissue from injected mice was resected at the time of harvest and RNA was isolated. IL-8 expression was measured in BOWES PCMV6 and CXCR3 tumor tissue via RT-PCR (**** = P≤ 0.0001). Representative individual tumors are presented in graph.

Overexpression of CXCR3 in human colon cancer cell lines and activation with ligands increases metastasis to the draining lymph nodes [24]. To further understand the consequences of CXCR3 expression in human melanoma cells and its role in the progression of BRAF^WT^ melanomas *in situ*, we orthotopically injected 5 x 10^5^ BOWES CXCR3 and BOWES PCMV6 cells intradermally in NSG mice (n = 16 mice per cell line). Detectable tumors arose earlier in mice injected with BOWES CXCR3 cells (7 out of 16 mice in 2 weeks) compared to those injected with BOWES PCMV6 cells (4 out of 16 mice in 3 weeks) (Table 2). For each week, the tumor volumes (mm^3^) were calculated only from mice that had tumors at that time. When these tumor volumes (mm^3^) were plotted over the 6 week growth period, the slope of the BOWES CXCR3 tumors was significantly steeper than that of the BOWES PCMV6 tumors (Fig. 5A, P = 0.006). Taken together, these data show that, compared to mice injected with BOWES PCMV6 cells, mice injected with BOWES CXCR3 cells had decreased tumor latency and increased tumor growth rate.

**Table 2:** Tumor growth *in vivo*.

** **	****PCMV6****		****CXCR3****		P-value
****Weeks post injection****	Frequency	Volume ± SEM	Frequency	Volume ± SEM	
**1**	0/16	0 ± 0	0/16	0 ± 0	NS
**2**	0/16	0 ± 0	7/16	74.4 ± 15.0	****
**3**	4/16	26.5 ± 8.5	15/16	131.3 ± 23.0	*
**4**	8/16	100.0 ± 19.2	16/16	176.1 ± 24.3	*
**5**	10/16	129.0 ± 32.7	16/16	253.1 ± 35.1	*
**6**	10/16	180.6 ± 36.4	16/16	323.7 ± 36.7	*

BOWES PCMV6 and CXCR3 cells were injected interdermally into NSG mice (5 x 10^5^ cells per injection). Tumors were measured weekly with calipers and tumor volume (mm^3^) was calculated. Mean tumor volume and SEM were calculated from mice that had measurable tumors. The table shows tumor incidence and the mean tumor volume (with SEM). T-tests were performed comparing tumor volumes of BOWES PCMV6 and CXCR3 injected mice at individual weeks (NS = P>0.5, * = P≤ 0.05, **** = P≤ 0.0001).

Six weeks after injections, when the tumors reached 8–10mm in diameter, mice were sacrificed and the draining lymph nodes (DLNs) were harvested from each mouse. Human DNA, due to metastases, was assessed by PCR detection of human *Alu* sequences [29,32]. In agreement with others, BOWES CXCR3 cells produced more metastases to the DLNs (6/13) than BOWES PCMV6 cells (1/13) (P = 0.037). These data are consistent with human colon cancer cells, which show significant increases in the metastases to the lymph nodes in CXCR3 overexpressing cells compared to empty vector controls [24], suggesting an association with CXCR3 and the RGP to VGP transition in melanoma cells. Given the upregulation of IL-8 expression in BOWES CXCR3 cells *in vitro* (Fig. 3E), we examined expression of IL-8 mRNA in tumor tissue. All BOWES CXCR3 tumors had significantly higher levels of IL-8 expression compared to BOWES PCMV6 tumors (Fig. 5B, P≤ 0.0001). Given that IL-8 mRNA is mirrored by an increase in IL-8 protein in human melanoma cells [29], our data are consistent with the observation that ectopic expression of IL-8 in RGP melanoma cells has been shown to increase their tumorigenicity and metastatic potential [20].

## Discussion

In this study, we demonstrate that signaling through CXCR3 induces IL-8 expression in a MAPK-dependent manner. Initially we show that *in vitro* environmental stress and nutrient deprivation, as defined by serum-free medium and increased C0_2_ levels, increase endogenous expression of CXCR3 on two human RGP cell lines, BOWES and WM35. BOWES is wild-type for the BRAF^V600E^ mutation, which is found in about 50% of melanoma, while WM35 contains the mutation. In BOWES cells, but not in WM35 cells, increased endogenous CXCR3 expression is linked with IL-8 expression.

Ectopic overexpression of CXCR3 in BOWES cells leads to increased ligand-mediated phosphorylation of ERK, cellular migration, and IL-8 expression *in vitro*, and to increased tumorigenesis, lymph node metastasis, and IL-8 expression *in vivo*. Our results suggest that, in BRAF^WT^ melanomas, increases in CXCR3 signaling mediates increases in IL-8 expression, and consequently, that CXCR3 may contribute to the transition from RGP to VGP in the melanoma patient population where the BRAF^V600E^ mutation is not present.

Tumor growth during progression exposes melanoma cells to increased cellular stress, hypoxia and nutrient deprivation, in the tumor microenvironment (TME) [27]. Increased expression of CXCR3 has been correlated with hypoxia and nutrient deprivation *in vitro* [24,28], suggesting that cells expressing CXCR3 can survive and grow in the less favorable microenvironments of advanced cancer. Our data showing increased endogenous CXCR3 under conditions of environmental stress *in vitro* support this correlation. The fact that only a sub-population of cells display this increase suggests variations among cells within the tumor [29] and/or that the TME may favor increased expression in one area of the tumor versus another, and thus facilitate the migration and escape of certain tumor cells from the primary tumor but not all cells.

Previous studies have demonstrated that knock down of constitutive expression of CXCR3 in a VGP murine melanoma cell line decreases metastatic frequency to adjacent lymph nodes [25]. In addition, exogenous expression of CXCR3 in a human colon cancer cell line, increased metastasis of these cells to the draining lymph nodes (DLNs) of injected mice, compared to non-expressing cells [24]. Our results confirm both studies by showing increased metastasis to DLNs in BOWES CXCR3 cells compared to BOWES PCMV6 cells. The CXCR3 ligands, CXCL9 and CXCL10, within the LNs of host mice have facilitated metastasis of murine melanoma cells through CXCR3 [25]. Given that we find mouse CXCL9 and CXCL10 are capable of inducing CXCR3 signaling in BOWES CXCR3 cells, in agreement with others [36], this suggests that the chemokines may be driving migration/metastasis to DLNs in these cells as well. However, in contrast to the finding that knocking down CXCR3 expression in murine melanoma has no effect on tumor size [25], we found that injection of BOWES CXCR3 cells decreased tumor latency and increased tumor growth compared to BOWES PCMV6 cells.

IL-8 (interleukin-8) was originally identified as a leukocyte chemoattractant, but has since been shown to be involved in melanoma progression, angiogenesis induction, and metastatic potential [33]. IL-8 has also been shown to increase haptotactic migration in melanoma cells across a membrane [46], which supports our *in vitro* findings that BOWES CXCR3 cells, with increased IL-8 expression, have increased migration toward CXCL9 and CXCL10, and our *in vivo* findings that BOWES CXCR3 tumor tissue, with increased IL-8 expression, have increased metastasis to DLNs. Nonetheless, studies have shown that the mouse IL-8 receptor does not bind human IL-8 in the nanomolar range [47], suggesting that increased expression of IL-8 found in BOWES CXCR3 tumor tissue is indirectly effecting the mouse TME.

One study found that ectopic expression of IL-8 in RGP melanoma cells increased their tumorigenicity and metastatic potential [20]. However, this study did not specify the BRAF mutation status of the cell line tested. We find higher levels of IL-8 expression in WM35 BRAF^V600E^ cells compared to BOWES BRAF^WT^ cells, consistent with the findings of other investigators, who report high IL-8 expression in the presence of the BRAF^V600E^ mutation [48]. In addition, we find that IL-8 expression in BOWES cells, but not WM35 cells, is mediated by CXCR3 signaling, and that this expression is dependent on phospho-ERK. These data suggest that, while CXCR3 signaling is able to mediate IL-8 expression in BRAF^WT^ melanoma, other mechanisms are regulating IL-8 expression in BRAF^V600E^ cell lines. Given the constitutive phosphorylation of ERK in BRAF^V600E^ melanoma cells, it is possible that the ERK-mediated IL-8 expression in these cells is already too saturated to be affected by CXCR3 signaling. Since RGP cell lines can harbor the BRAF^V600E^ mutation and express substantial levels of IL-8, other mechanisms may contribute to VGP transition of BRAF^V600E^ cell lines, such as WM35. In contrast, our study directly links CXCR3 signaling with IL-8 expression in BRAF^WT^ melanoma cells, and suggests a role for CXCR3 in melanoma progression in human cells that do not harbor the BRAF^V600E^ mutation.

## Supporting Information

S1 Fig CXCR3 expression on human VGP melanoma cell lines. (TIF)

S2 Fig IL-8 and CXCL10 expression in stressed BOWES cells without ligands. (TIF)

S3 Fig BOWES CXCR3 cells stained with IgG1 isotype control and CXCR3 antibodies. (TIF)

S1 Table Real Time RT-PCR primer sequences. (DOC)
