# Supplementary figures and images for: CXCR3 Signaling in BRAFWT Melanoma Increases IL-8 Expression and Tumorigenicity
Source: PLoS One. 2015 Mar 23;10(3):e0121140. doi: 10.1371/journal.pone.0121140 (PMC4370421; doi:10.1371/journal.pone.0121140)

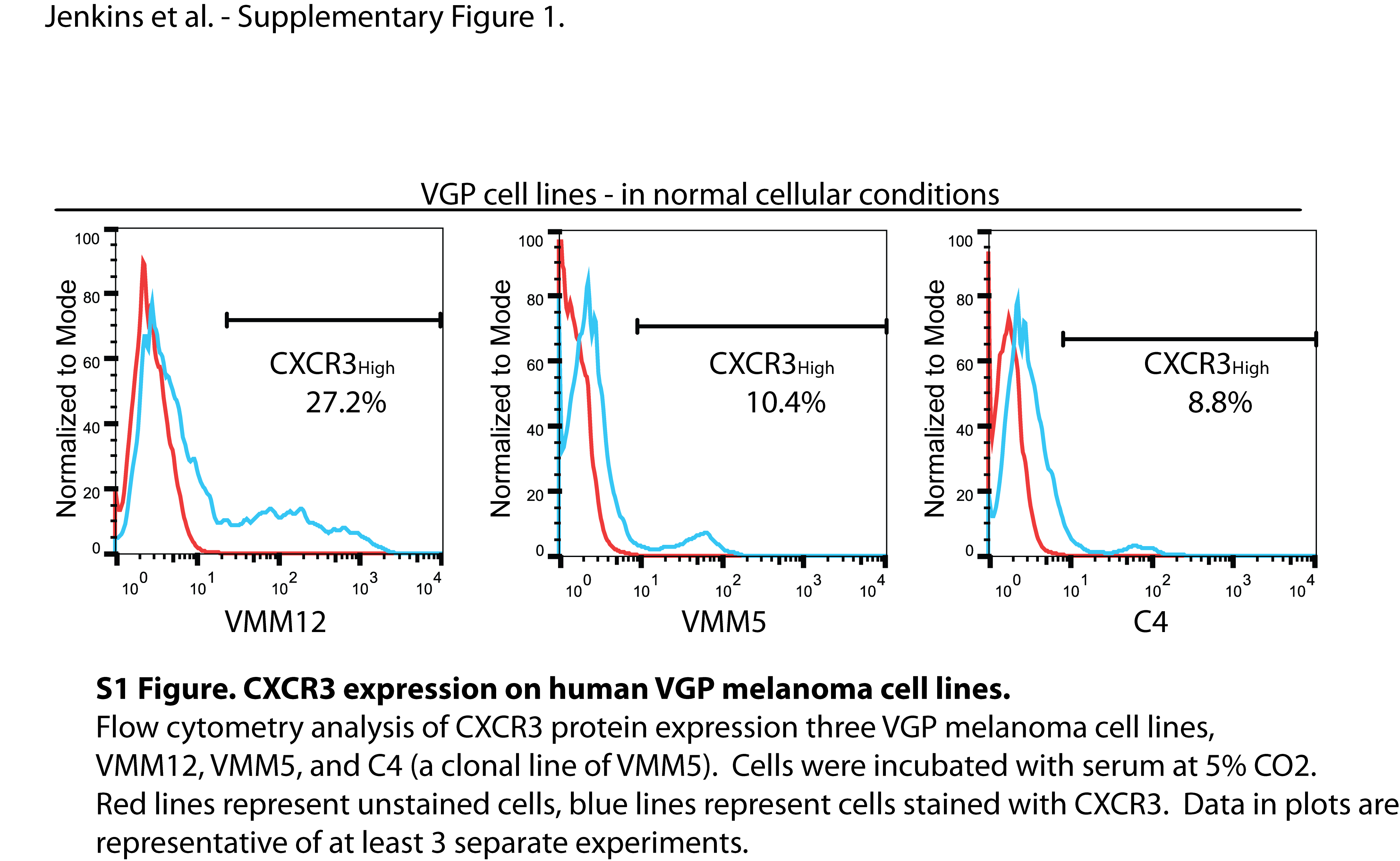

Supplement: S1 Fig — (TIF) [file pone.0121140.s001.tif]

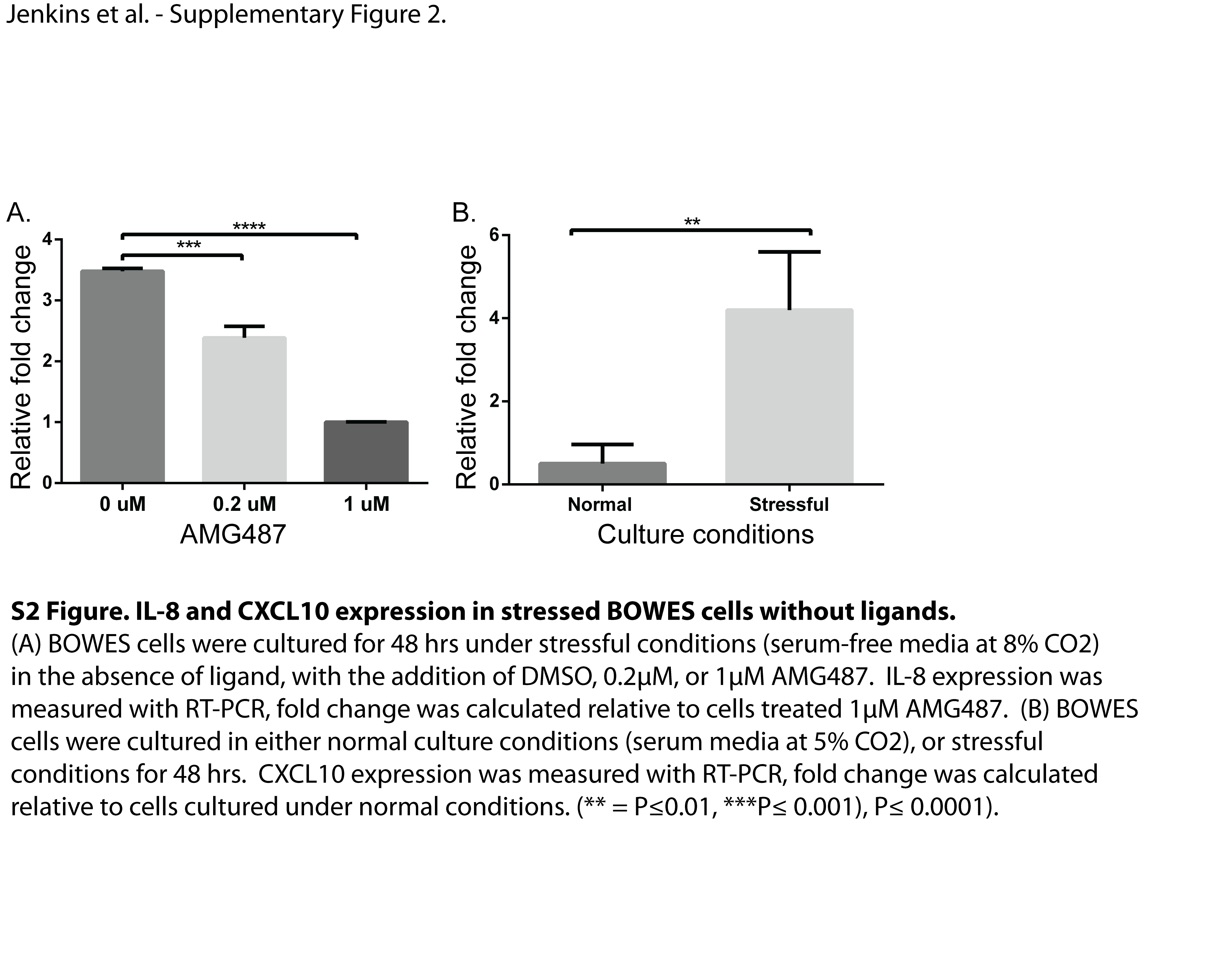

Supplement: S2 Fig — (TIF) [file pone.0121140.s002.tif]

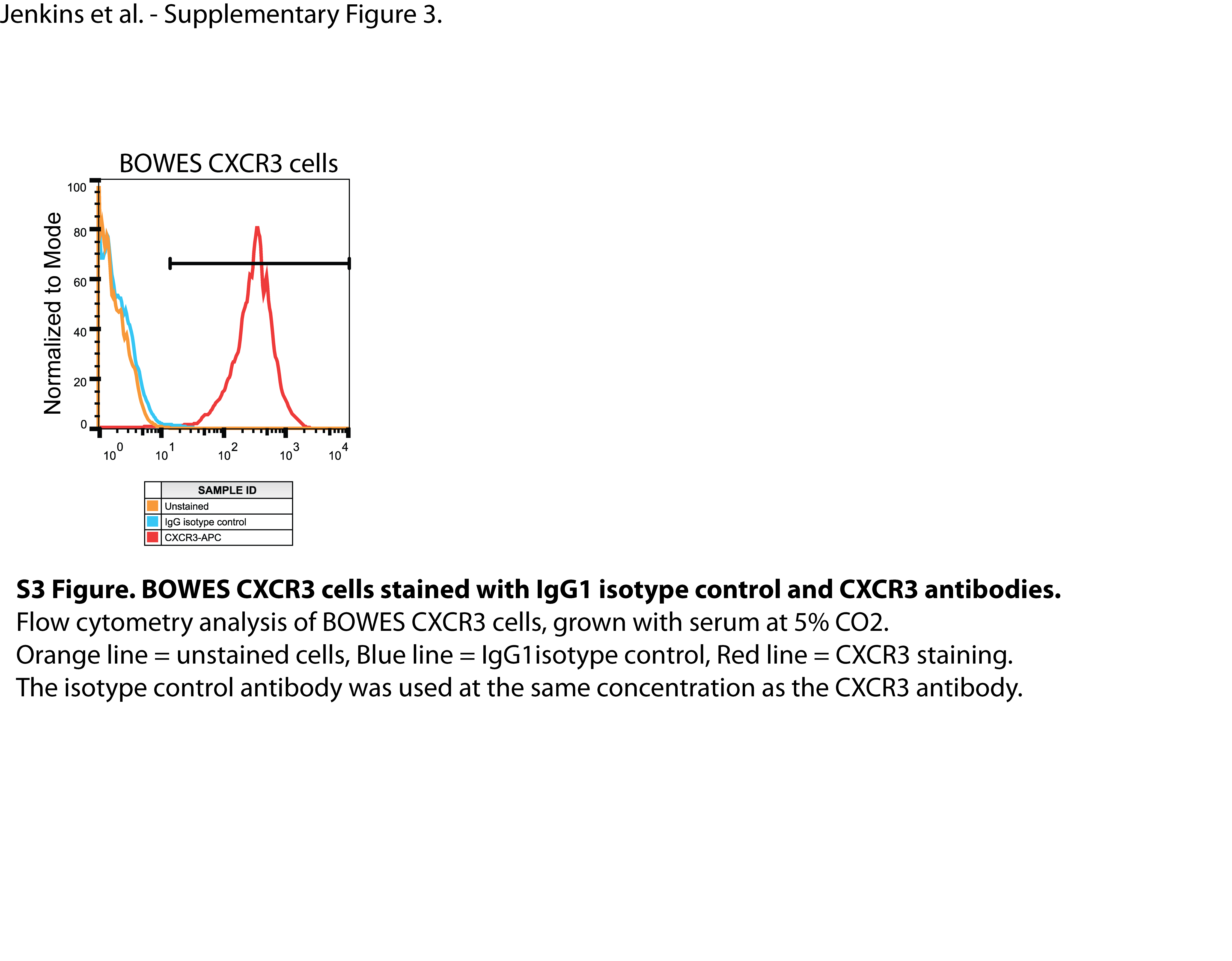

Supplement: S3 Fig — (TIF) [file pone.0121140.s003.tif]
