# Supplementary material for: CXCR3 Signaling in BRAFWT Melanoma Increases IL-8 Expression and Tumorigenicity
Source: PLoS One. 2015 Mar 23;10(3):e0121140. doi: 10.1371/journal.pone.0121140 (PMC4370421; doi:10.1371/journal.pone.0121140)
Supplement: S1 Table — (DOC) [file pone.0121140.s004.doc]

Supplementary Table 1: Real Time RT-PCR Primer Sequences

|  | | |
| --- | --- | --- |
| **Gene Name** | **Primer Direction** | **Sequence** |
| **B2M** | Forward | TGCTGTCTCCATGTTTGATGTATCT |
|  | Reverse | TCTCTGCTCCCCACCTCTAAGT |
| **HIF-1alpha** | Forward | TTCACCTGAGCCTAATAGTCC |
|  | Reverse | CAAGTCTAAATCTGTGTCCTG |
| **IL-1beta** | Forward | ACCCTCTGTCATTCGCTCCCACA |
|  | Reverse | AGAGCACACCAGTCCAAATTGAATTG |
| **IL-6** | Forward | TCGAGCCCACCGGGAACGAA |
|  | Reverse | GCAACTGGACCGAAGGCGCT |
| **IL-8** | Forward | GAGTGGACCACACTGCGCCAA |
|  | Reverse | TCCACAACCCTCTGCACCCAGTT |
| **MCP-1** | Forward | TTCATTCCCCAAGGGCTCGCT |
|  | Reverse | GGGTTTGCTTGTCCAGGTGGTC |
| **Par-1** | Forward | ACCCGCAGAAGTCAGGAG |
|  | Reverse | CCGGGGATCTAAGGTGGCATTTGT |
| **MMP-1** | Forward | AGCTAGCTCAGGATGACATTGATG |
|  | Reverse | GCCGATGGGCTGGACAG |
| **MMP-2** | Forward | TGGCGATGGATACCCCTTT |
|  | Reverse | TCCTCCCAAGGTCCATAGCTCAT |
| **MMP-3** | Forward | TTCCGCCTGTCTCAAGATGATAT |
|  | Reverse | AAAGGACAAAGCAGGATCACAGTT |
| **MMP-9** | Forward | CCTTGTGCTCTTCCCTGG AGACC |
|  | Reverse | GGCCTTCAGCGTGGCGCTATC |
| **MMP-13** | Forward | TCTTGACCACTCCAAGGACC |
|  | Reverse | AAGGGTCACATTTGTCTGGC |
| **MMP-14** | Forward | CCCCGAAGCCTGGCTAGA |
|  | Reverse | GCATCAGCTTTGCCTGTTACT |
| **mouse B2M** | Forward | GCCGAACATACTGAACTGCTAC |
|  | Reverse | GGCCATACTGTCATGCTTAACT |
| **ALU Yb8** | Forward | CGAGGCGGGTGGATCATGAGGT |
|  | Reverse | TCTGTCGCCCAGGCCGGACT |
